# Mechanics and energetics of post-stroke walking aided by a powered ankle exoskeleton with speed-adaptive myoelectric control

**DOI:** 10.1186/s12984-019-0523-y

**Published:** 2019-05-15

**Authors:** Emily M. McCain, Taylor J. M. Dick, Tracy N. Giest, Richard W. Nuckols, Michael D. Lewek, Katherine R. Saul, Gregory S. Sawicki

**Affiliations:** 1 North Carolina State University, 911 Oval Drive, Raleigh, NC 27606 USA; 2 School of Biomedical Sciences, University of Queensland, St. Lucia, QLD Australia; 3 Harvard University, Cambridge, MA USA; 4 University of North Carolina at Chapel Hill, Chapel Hill, NC USA; 5 Georgia Institute of Technology, Atlanta, GA USA

**Keywords:** Ankle mechanics, Exoskeleton, Trailing limb angle, Propulsion, Myoelectric control, Electromyography, Metabolic cost, Hemiparesis, Stroke rehabilitation, Walking

## Abstract

**Background:**

Ankle exoskeletons offer a promising opportunity to offset mechanical deficits after stroke by applying the needed torque at the paretic ankle. Because joint torque is related to gait speed, it is important to consider the user’s gait speed when determining the magnitude of assistive joint torque. We developed and tested a novel exoskeleton controller for delivering propulsive assistance which modulates exoskeleton torque magnitude based on both soleus muscle activity and walking speed. The purpose of this research is to assess the impact of the resulting exoskeleton assistance on post-stroke walking performance across a range of walking speeds.

**Methods:**

Six participants with stroke walked with and without assistance applied to a powered ankle exoskeleton on the paretic limb. Walking speed started at 60% of their comfortable overground speed and was increased each minute (n00, n01, n02, etc.). We measured lower limb joint and limb powers, metabolic cost of transport, paretic and non-paretic limb propulsion, and trailing limb angle.

**Results:**

Exoskeleton assistance increased with walking speed, verifying the speed-adaptive nature of the controller. Both paretic ankle joint power and total limb power increased significantly with exoskeleton assistance at six walking speeds (n00, n01, n02, n03, n04, n05). Despite these joint- and limb-level benefits associated with exoskeleton assistance, no subject averaged metabolic benefits were evident when compared to the unassisted condition. Both paretic trailing limb angle and integrated anterior paretic ground reaction forces were reduced with assistance applied as compared to no assistance at four speeds (n00, n01, n02, n03).

**Conclusions:**

Our results suggest that despite appropriate scaling of ankle assistance by the exoskeleton controller, suboptimal limb posture limited the conversion of exoskeleton assistance into forward propulsion. Future studies could include biofeedback or verbal cues to guide users into limb configurations that encourage the conversion of mechanical power at the ankle to forward propulsion.

**Trial registration:**

N/A.

**Electronic supplementary material:**

The online version of this article (10.1186/s12984-019-0523-y) contains supplementary material, which is available to authorized users.

## Background

Walking after a stroke is more metabolically expensive, leading to rapid exhaustion, limited mobility, and reduced physical activity [1]. Hemiparetic walking is slow and asymmetric compared to unimpaired gait. Preferred walking speeds following stroke range between < 0.2 m s^− 1^ and ~ 0.8 m s^− 1^ [2] compared to ~ 1.4 m s^− 1^ in unimpaired adults, and large interlimb asymmetry has been documented in ankle joint power output [3, 4]. The ankle plantarflexors are responsible for up to 50% of the total positive work needed to maintain forward gait [5, 6]; therefore, weakness of the paretic plantarflexors is especially debilitating, and as a result, the paretic ankle is often a specific target of stroke rehabilitation [7–10]. In recent years, ankle exoskeletons have emerged as a technology capable of improving ankle power output by applying torque at the ankle joint during walking in clinical populations [7, 8] and healthy controls [11–14]. Myoelectric exoskeletons offer a user-controlled approach to stroke rehabilitation by measuring and adapting to changes in the user’s soleus electromyography (EMG) when generating torque profiles applied at the ankle [15]. For example, a proportional myoelectric ankle exoskeleton was shown to increase the paretic plantarflexion moment for persons post-stroke walking at 75% of their comfortable overground (OVG) speed [8]; despite these improvements, assistance did not reduce the metabolic cost of walking or improve percent paretic propulsion. The authors suggested exoskeleton performance could be limited because the walking speed was restricted to a pace at which exoskeleton assistance was not needed.

Exoskeleton design for improved function following a stroke would benefit from understanding the interaction among exoskeleton assistance, changes in walking speed, and measured walking performance. Increases in walking speed post-stroke are associated with improvements in forward propulsion and propulsion symmetry [16], trailing limb posture [17, 18], step length symmetries [17, 19], and greater walking economies [17, 19]. This suggests that assistive technologies need to account for variability in walking speeds to further improve post-stroke walking outcomes. However, research to date has evaluated exoskeleton performance at only one walking speed, typically set to either the participant’s comfortable OVG speed or a speed below this value [7, 8]. At constant speeds, ankle exoskeletons have been shown to improve total ankle power in both healthy controls [11] and persons post-stroke [8], suggesting the joint powers and joint power symmetries could be improved by exoskeleton technology. Additionally, an exosuit applying assistance to the ankle was able to improve paretic propulsion and metabolic cost in persons post-stroke walking at their comfortable OVG speed [7]. Assessing the impact of exoskeleton assistance on walking performance across a range of speeds is the next logical step toward developing exoskeleton intervention strategies targeted at improving walking performance and quality of life for millions of persons post-stroke.

In order to assess the impact of exoskeleton assistance across a range of walking speeds in persons post-stroke, we developed a novel, speed-adaptive exoskeleton controller that automatically modulates the magnitude of ankle torque with changes in walking speed and soleus EMG. We hypothesized that: 1) Our novel speed-adaptive controller will scale exoskeleton assistance with increases in walking speed as intended. 2) Exoskeleton assistance will lead to increases in total average net paretic ankle power and limb power at all walking speeds. 3) Exoskeleton assistance will lead to metabolic benefits associated with improved paretic average net ankle and limb powers.

## Methods

### Exoskeleton hardware

We implemented an exoskeleton emulator comprised of a powerful off-board actuation and control system, a flexible Bowden cable transmission, and a lightweight exoskeleton end effector [20]. The exoskeleton end effector includes shank and foot carbon fiber components custom fitted to participants and hinged at the ankle. The desired exoskeleton torque profile was applied by a benchtop motor (Baldor Electric Co, USA) to the carbon-fiber ankle exoskeleton through a Bowden-cable transmission system. An inline tensile load cell (DCE-2500 N, LCM Systems, Newport, UK) was used to confirm the force transmitted by the exoskeleton emulator during exoskeleton assistance.

### Speed-adaptive proportional myoelectric exoskeleton controller

**Fig. 1:**
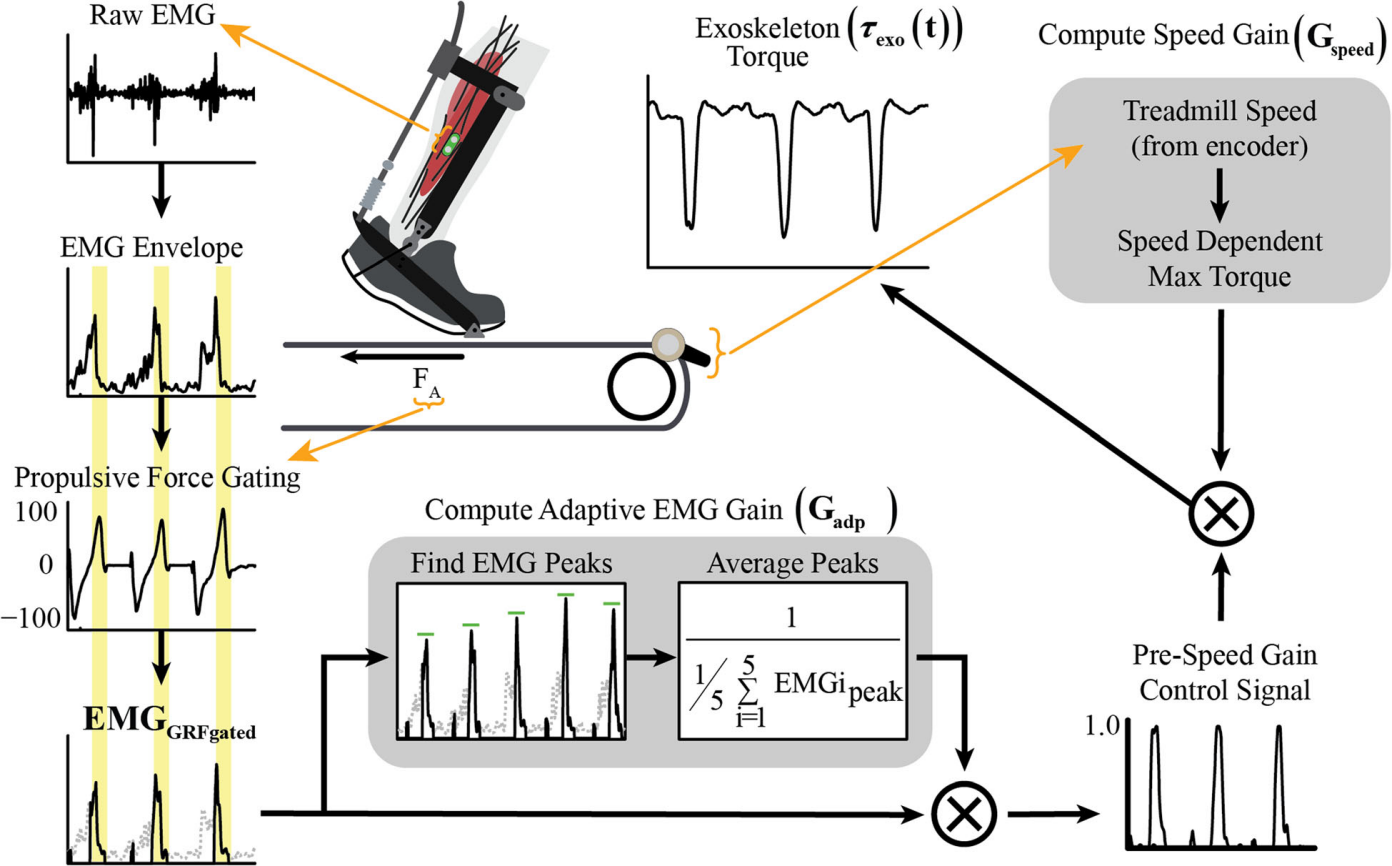
Novel speed-adaptive myoelectric exoskeleton controller measures and adapts to users’ soleus EMG signal as well as their walking speed in order to generate the exoskeleton torque profile. Raw soleus EMG signal is filtered and rectified to create an EMG envelope, and the created EMG envelope is then gated by anterior GRFs to ensure assistance is only applied during forward propulsion. The adaptive EMG gain is calculated as a moving average of peak force-gated EMG from the last five paretic gait cycles. The pre-speed gain control signal is the product of the force-gated EMG and the adaptive EMG gain. The speed gain is determined using real-time walking speed and computed as 25% of the maximum biological plantarflexion torque at that given walking speed. Exoskeleton torque is the result of multiplying the speed gain with the pre-speed gain control signal

Our exoskeleton controller alters the timing and magnitude of assistance with the user’s soleus EMG signal and walking speed (Fig. 1). The exoskeleton torque is determined from Eq. 1, in which participant mass (m_participant_) is constant across speeds, treadmill speed (V) is measured in real-time, the speed gain (G_speed_) is constant for all subjects and across speeds, the adaptive gain (G_adp_) is constant for a gait cycle and calculated anew for each gait cycle, and the force-gated and normalized EMG (EMG_GRFgated_) is a continuously changing variable.

1 $$ {\tau}_{exo}\ (t)={m}_{participant}\times V\times {G}_{speed}\times {G}_{adp}\times {EMG}_{GRFgated} $$

Surface EMG was collected for the paretic soleus at 960 Hz (SX230, Biometrics, Newport, UK), high-pass filtered with a 2nd order dual-pass Butterworth filter (50 Hz), full-wave rectified, low-pass filtered with 2nd order dual-pass Butterworth filter (10 Hz) and normalized to one by the adaptive gain [15]. In persons post-stroke, spasticity, altered coordination, and weakness [21] can affect soleus activation timing and magnitude. In order to maintain volitional control while ensuring exoskeleton torque was only applied during forward propulsion, the EMG envelope was gated by anterior ground reaction forces (GRFs) [8]. Our adaptive EMG gain (G_adp_) was calculated as the inverse of the moving average of the peak of the force-gated EMG envelope from the previous five gait cycles. Vertical GRFs were used to determine heel strikes. The EMG adaptive gain multiplied by the force-gated EMG signal produces the pre-speed control signal allowing the shape of the EMG envelope to be maintained, with the peak normalized to one. The speed-adaptive gain (G_speed_) was determined empirically from pilot data to scale the pre-speed control signal to ~ 25% of the maximum normal biological ankle plantarflexion moment as predicted from normative data relating peak plantarflexion given body mass and treadmill velocity; the speed gain has units of (N m (m s^− 1^)^− 1^) kg^− 1^. The participant’s real-time walking speed and mass are multiplied by the speed gain and the pre-speed control signal to determine an exoskeleton torque in Newton-meters. Applying ~ 25% of normal biological ankle plantarflexion moment ensures the torque applied by our controller is comparable to that applied by previous ankle exoskeletons [8, 11]. Instantaneous treadmill velocity was recorded by a speed encoder (1024cpr, Encoder Products Company, USA) secured to the split belt treadmill roller (Bertec, USA).

### Inclusion criteria

Participants were required to be at least 6 months post-stroke and to demonstrate persistent lower extremity hemiparesis with a comfortable OVG walking speed of at least 0.6 m s^− 1^ and the ability to walk on a treadmill for at least 5 min at a time.

### Data collection

Data collection procedures were approved by the University of North Carolina at Chapel Hill institutional review board (IRB), and all participants signed an IRB approved consent form before data collections. Experimental data were collected from six persons post-stroke (Table 1) walking on an instrumented split belt treadmill (Bertec, USA): (1) wearing the exoskeleton on the paretic ankle, but without powered assistance (*Unassisted*) and (2) wearing the exoskeleton as it provided powered assistance (*Assisted*). Each session was performed on a separate day, and conditions were counter-balanced. Participants started by walking at 60% of their preferred speed (n00). At each consecutive minute, the treadmill speed was increased by 0.1 m s^− 1^ (n01, n02, etc) until the subject reached one of several stopping criteria (heart rate reached 60% of their heart rate reserve; rate of perceived exertion exceeds 7 (on a Borg 1–10 scale); or the subject asked to stop). Preferred OVG walking speed was assessed over a 10 m overground walkway. No body weight support was provided; however, all participants wore a harness for fall prevention. Use of handrails mounted bilaterally was discouraged.

**Table 1:** Subject Characteristicss

Participant	Gender	Affected Side	Age (yrs)	Mass (Kg)	Height (m)	Months Since Stroke	OVG Speed (ms^−1^)
1	F	L	47	80.8	1.7	151.0	0.83
2	M	R	50	71.3	1.7	41.0	1.02
3	M	R	56	90.2	1.9	19.0	0.82
4	F	R	43	98.3	1.6	23.0	0.84
5	F	L	40	70.9	1.6	33.0	0.60
6	M	R	62	91.5	1.9	180.0	1.00
Average		–	49.7	83.8	1.7	74.5	0.85
Std Dev		–	8.2	11.3	0.1	71.5	0.15

An eight-camera motion analysis system (Vicon, Oxford, UK) recorded positions of 37 reflective markers attached to the pelvis and legs (modified Cleveland Clinic marker set, similar to [22]) at 120 Hz. The modified marker set consisted of 26 anatomical markers placed over: the greater trochanter, illiac crest, lateral femoral epicondyle, medial femoral epicondyle, lateral malleolus, medial malleolus, calcaneus, and second metatarsophalangeal joint of both limbs. The remaining markers were placed in clusters of three or four on the pelvis, feet, thigh and shank segments. The foot clusters were attached to each participant’s shoes. Raw marker positions were filtered using a second order low-pass Butterworth filter (cut-off frequency of 10 Hz). Anatomical markers from a static standing collection were used to scale and calibrate segments (pelvis, thighs, shanks and feet) for each participant; inertial properties were applied to scaled and calibrated segments, and default geometries used to create subject specific models (Visual 3D, C-Motion, USA). A second order low-pass Butterworth filter with a cutoff frequency of 40 Hz was applied to raw analog force platform signals. Rates of oxygen consumption and carbon dioxide production were recorded on a breath-by-breath basis using a portable metabolic system (OxyCon Mobile, Carefusion, USA). To obtain baseline metabolic energy consumption during standing, measurements were made during 5 min of quiet standing prior to speed ramp sessions.

### Data processing

Detailed descriptions of the analyses used in this investigation have been provided previously [8, 22]. Briefly, an inverse kinematics algorithm [23] was used to obtain ankle, knee, and hip joint angles processed in Visual3D (CMotion, USA) and MATLAB (Mathworks, USA) from filtered marker data and individual models. An inverse dynamics algorithm was used to determine joint moments and powers. To evaluate walking performance with and without the exoskeleton at different speeds, we obtained measures of exoskeleton assistance from a load cell within the device.

Exoskeleton assistance, joint and limb powers, integrated anterior GRFs, and trailing limb angle (TLA) were calculated as an average over paretic and non-paretic gait cycles in the five analyzed strides. Analyzed strides occurred during the latter half of each minute to allow for adjustment before and after changes in treadmill speed. Crossover steps were excluded from analysis. If a subject did not complete five strides at a speed before reaching the stopping criteria, the speed was not included in this study. At higher speeds, the sample size decreased as some participants reached the stopping criteria. Metabolic cost of transport was calculated for each subject as the total cost for the entire session over all recorded speeds.

#### Exoskeleton assistance

For *Assisted* conditions we determined exoskeleton torque about the ankle by multiplying the measured exoskeleton force from the in-series load cell by the moment arm, determined as the measured linear distance between the ankle joint center and the exoskeleton cable in a neutral position. Biological torque at the ankle was calculated as the difference between the total ankle torque calculated from inverse dynamics and the applied exoskeleton torque. Multiplying torque and ankle joint angular velocity yielded the exoskeleton mechanical power contribution in watts (W) [23].

#### Average joint power

We calculated average positive, average negative, and average net mechanical power for the ankle, knee, and hip joints and the exoskeleton. Calculations of average joint powers have been described previously [22]. Briefly, positive and negative intervals of time series joint powers were separately integrated with time to determine total positive and negative work done. Positive and negative work were divided by the sum of the associated intervals of time to determine average positive and negative powers for a gait cycle. Average net power was determined from the integral of time series joint powers divided by the duration of the five integrated strides.

#### Average limb power

Calculations for average positive, average negative, and average net limb powers have been described previously [22]. In brief, time series joint power curves were summed for each of the paretic and non-paretic limbs to yield limb power with time. Time series limb power was integrated to determine net work done. Net work was divided by the sum of the associated stride times to determine net power. Total positive and negative work done by the limb was determined by separately integrating positive and negative integrals of time series limb power. Limb powers were determined by dividing work by the associated time integrals from the five strides.

#### Net metabolic power and metabolic cost of transport

We used a portable metabolic system to collect rates of oxygen consumption and carbon dioxide production during all data sessions as input into the Brockway equation to calculate metabolic power (W) [24]. Prior to walking, data from the last 2 min of 5 min quiet standing were averaged and used to determine metabolic power during standing. Net metabolic power was calculated by subtracting metabolic power during standing (W) from metabolic power during walking (W) and then normalized to individual body mass (kg). For both the *Assisted* and *Unassisted* data collection sessions, we integrated net metabolic power (W kg^− 1^) to determine energy consumed (J kg^− 1^) during each session. We then divided energy by the total distance traveled (m) during the walking session to calculate net metabolic cost of transport for the session (J m^− 1^ kg^− 1^).

#### Paretic and non-paretic propulsion

Intervals of anteriorly directed GRFs were trapezoidally integrated with time over five gait cycles for the paretic and non-paretic limbs. Subject average paretic and non-paretic propulsion were calculated for each speed as well as the comfortable OVG speed [25].

#### Peak vertical ground reaction force during propulsion

Peak GRFs occuring during periods of forward propulsion were identified as the second peak in vertical GRFS for five gait cycles on the paretic limb and normalized by body weight for each subject and at each speed [26, 27]. Peak values were averaged across gait cycles and across speeds.

#### TLA

TLA was defined in the sagittal plane as the maximum angle between the vertical axis and a line connecting the greater trochanter with the second metatarsophalangeal joint during double stance. Paretic and non-paretic double stances were defined between non-paretic heel strike and paretic toe off and between paretic heel strike and non-paretic toe off, respectively. Raw data were used to find paretic TLA at each time frame, and the maximum TLA was averaged across all paretic gait cycles and across speeds.

#### Statistical analyses

Differences between *Assisted* and *Unassisted* conditions for each subject for peak average ankle power, joint powers, limb powers, integrated anterior GRFs, and TLA were evaluated using paired t-tests (*α* = 0.05) and effect sizes (Cohen’s d) at each speed. Additionally, analysis of metabolic cost of transport included a paired t-test (*α* = 0.05) to determine differences between the *Assisted* and *Unassisted* conditions measured across all speeds. Effect sizes (d) were calculated by dividing the mean difference by the pooled standard deviation [28].

## Results

### Exoskeleton technology

**Fig. 2:**
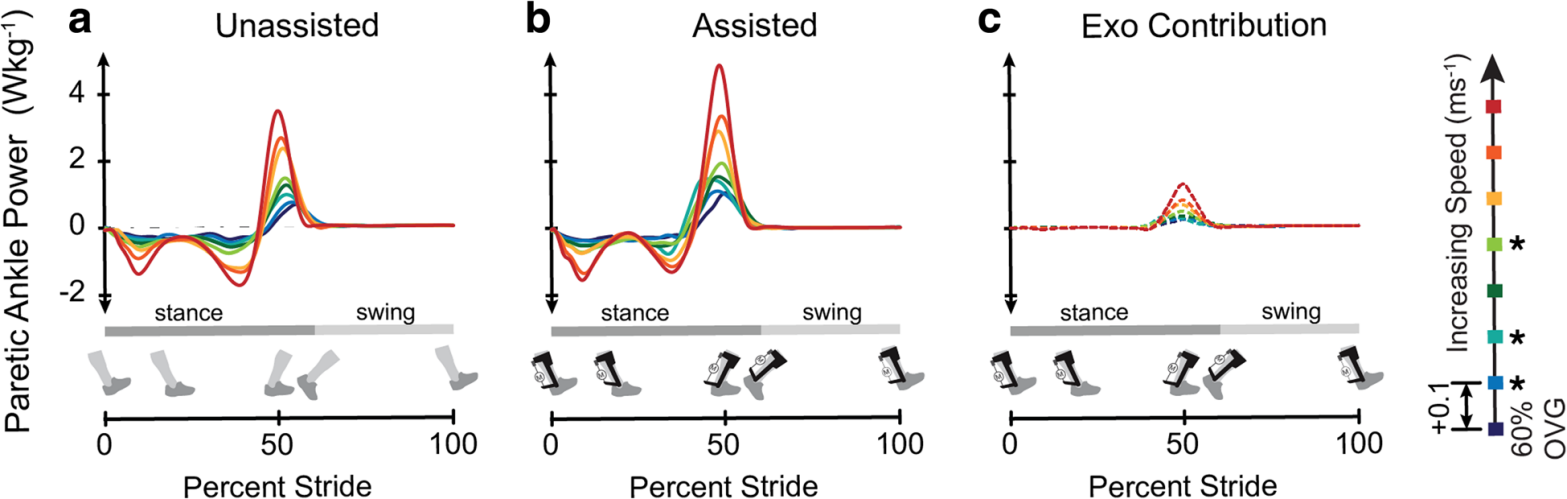
Peak paretic ankle power increased with walking speed and with exoskeleton assistance. Group average time-varying paretic ankle power in the *Unassisted* condition (**a**) and the *Assisted* condition (**b**), with the exoskeleton contribution isolated (**c**). Walking speed was increased from 60% of the users’ comfortable OVG speed (OVG) by 0.1 ms^−1^ each minute

The speed-adaptive proportional myoelectric exoskeleton controller increased peak assistance with speed, verifying the effectiveness of the speed-adaptive gain (Fig. 2c). Peak exoskeleton assistance ranged between 0.216 ± 0.097 N m^− 1^ kg^− 1^ and 0.354 ± 0.018 N m^− 1^ kg^− 1^, and peak assistance occurred with timing ranging from 43.6% ± 0.1% of stride to 49.3% ± 3.0% of stride (Table 2, Additional file 3: Figure S3). Peak total (biological + exoskeleton) paretic ankle power increased with exoskeleton assistance (Fig. 2b) when compared to the *Unassisted* condition (Fig 2a) at all speeds with significance at three of the eight speeds (*n01*: *p* = 0.002, *d* = 2.46; *n02*: *p* = 0.047, *d* = 1.71; *n04*: *p* = .015, *d* = 1.19). There was no significant change detected for peak ankle power in the non-paretic limb between the *Assisted* and *Unassisted* conditions (Additional file 4: Figure S4).

**Table 2:** Exoskeleton Peak Torque Timing and Magnitude with Walking Speed Sample Size

Speed	Peak Torque (Nm^−1^ kg^−1^)		Timing of Peak Torque (% stride)		Sample Size	
	Average	Std Dev	Average	Std Dev	*Unassisted*	*Assisted*
n00	0.216	0.097	49.3%	3.0%	6	6
n01	0.267	0.044	46.6%	1.4%	6	5*
n02	0.247	0.095	44.9%	1.8%	6	6
n03	0.276	0.048	45.8%	1.6%	6	6
n04	0.290	0.051	44.1%	2.5%	5	6
n05	0.352	0.014	43.7%	2.1%	3	3
n06	0.338	0.011	44.1%	0.6%	3	2
n07	0.354	0.018	43.6%	0.1%	2	2
OVG	n/a	n/a	n/a	n/a	6	6

### Joint mechanics

**Fig. 3:**
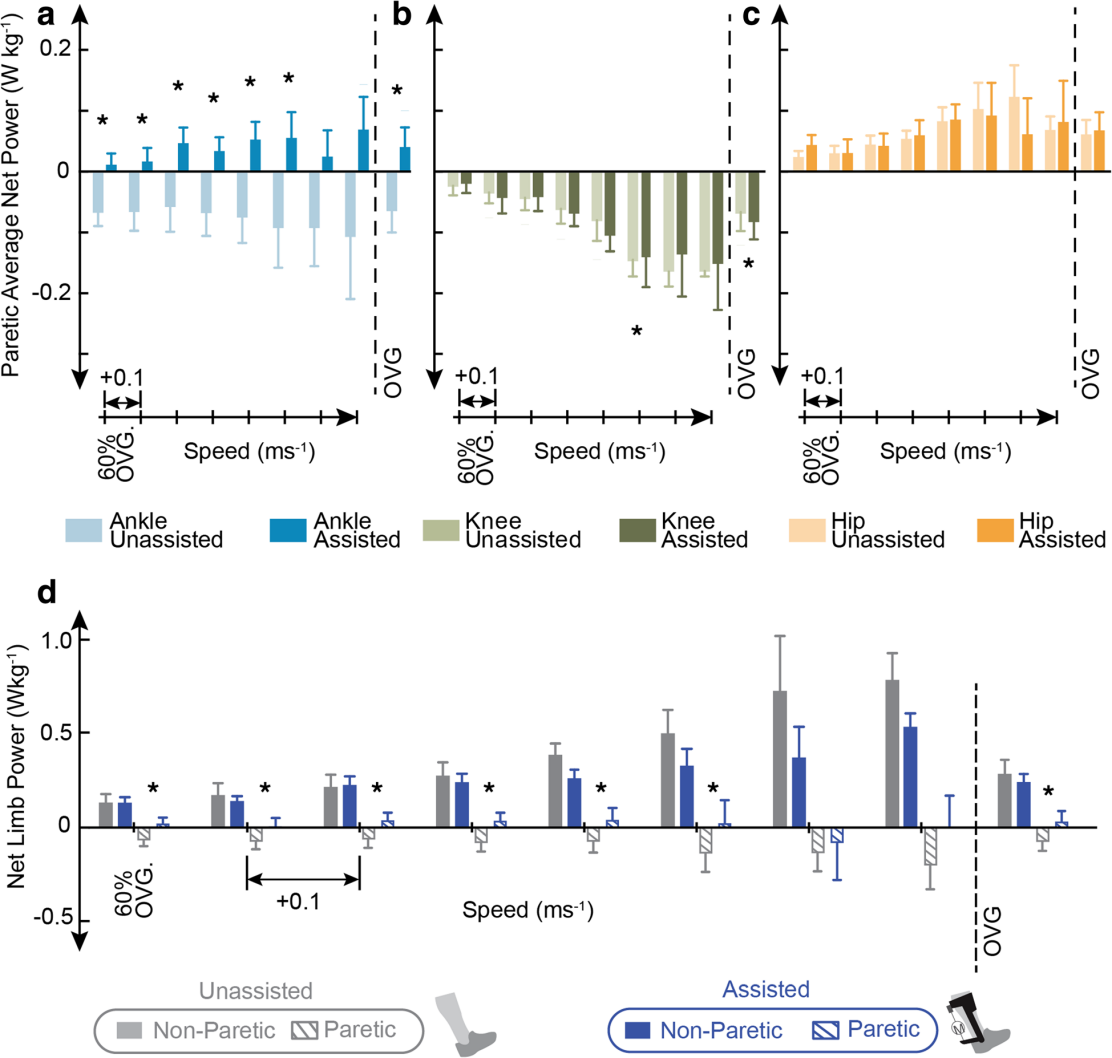
Average net paretic ankle and limb powers increased with exoskeleton assistance at all speeds. Average net paretic ankle (**a**), knee (**b**), and hip (**c**) power (± standard error) for the *Unassisted* (light colors) and *Assisted* (dark colors) conditions. Average net limb power (± standard error) for the paretic (hatch fill) and non-paretic (solid fill) limb with exoskeleton (blue) and without exoskeleton (grey) assistance (**d**). All values are calculated from subject averages over five gait cycles. To the right of the dashed line average net powers averaged at each user’s comfortable OVG speed are shown

Average net total paretic ankle power increased with assistance when compared to the *Unassisted* condition at six speeds (*n00: p* = 0.021, *d* = 1.40; *n01: p* = 0.008, *d* = 1.23; *n02: p* = 0.004, *d* = 1.29; *n03: p* = 0.003, *d* = 1.35; *n04: p* = 0.001, *d* = 1.56; *n05: p* = 0.013, *d* = 1.60) (Fig. 3a) and at each users’ preferred OVG speed (*p* = 0.003, *d* = 1.26). Average net paretic knee power decreased significantly in the *Assisted* condition at one speed (*n05*: *p* = 0.020, *d* = 0.51) and increased significantly at each users’ preferred OVG speed (*p* = 0.007, *d* = 0.20). No significant change was found in average net paretic hip power. Average positive and negative paretic joint powers were also calculated (Additional file 5: Figure S5 and Additional file 6: Figure S6).

No significant change was found in non-paretic average net ankle or hip power at any speed (Additional file 7: Figure S7). However, a significant decrease in average net knee power with exoskeleton assistance was seen at three speeds (*n00: p* = 0.045, *d* = 0.50; *n04: p* = 0.030, *d* = 0.60; *OVG: p* = 0.014, *d* = 0.60) (Additional file 7: Figure S7). Non-paretic average positive and negative joint powers were also calculated (Additional file 5: Figure S5 and Additional file 6: Figure S6).

### Limb mechanics

Average net paretic limb power increased with exoskeleton assistance at all speeds and with significance at seven speeds (*n00: p* = 0.010, *d* = 0.91; *n01: p* = 0.026, *d* = 0.60; *n02: p* = 0.0003, *d* = 0.80; *n03: p* = 0.002, *d* = 0.92; *n04: p* = 0.006, *d* = 0.65; *n05: p* = 0.035, *d* = 0.75; *OVG: p* = 0.007, *d* = 0.70). Average net non-paretic limb power was not significantly altered at any speed with exoskeleton assistance (Fig. 3d).

### Metabolics

Despite improvements in average net joint and limb powers on the paretic limb, we observed no significant change in the whole-body metabolic cost of transport with exoskeleton assistance (Table 3). Further, the impact of exoskeleton assistance on metabolic cost of transport was not consistent across individuals; with only two out of six participants experiencing a metabolic benefit with exoskeleton assistance (Table 3: Participant 4, Participant 6) (Additional file 8: Figure S8), and the remaining four participants displaying an increased cost of transport. Breath-by-breath data informing these calculations are included in supplemental materials (Additional file 8: Figure S8).

**Table 3:** Whole Body Metabolic Cost of Transport and Total Distance Traveled

Participants	Metabolic Cost of Tran sport ∫*Wkg*^−1^/Total Distance		Total Distance Walked (m)	
	Unassisted	Assisted	Unassisted	Assisted
1	3.2	3.6	188.1	172.8
2	3.4	3.6	435.2	452.5
3	3.8	4.2	417.2	493.4
4	3.0	2.9	163.9	135.7
5	2.8	3.7	127.6	160.5
6	4.2	3.7	331.7	324.8
Average	3.4	3.6	277.3	290.0
Std Dev	0.5	0.4	134.7	157.0

### Paretic and non-paretic propulsion

**Fig. 4:**
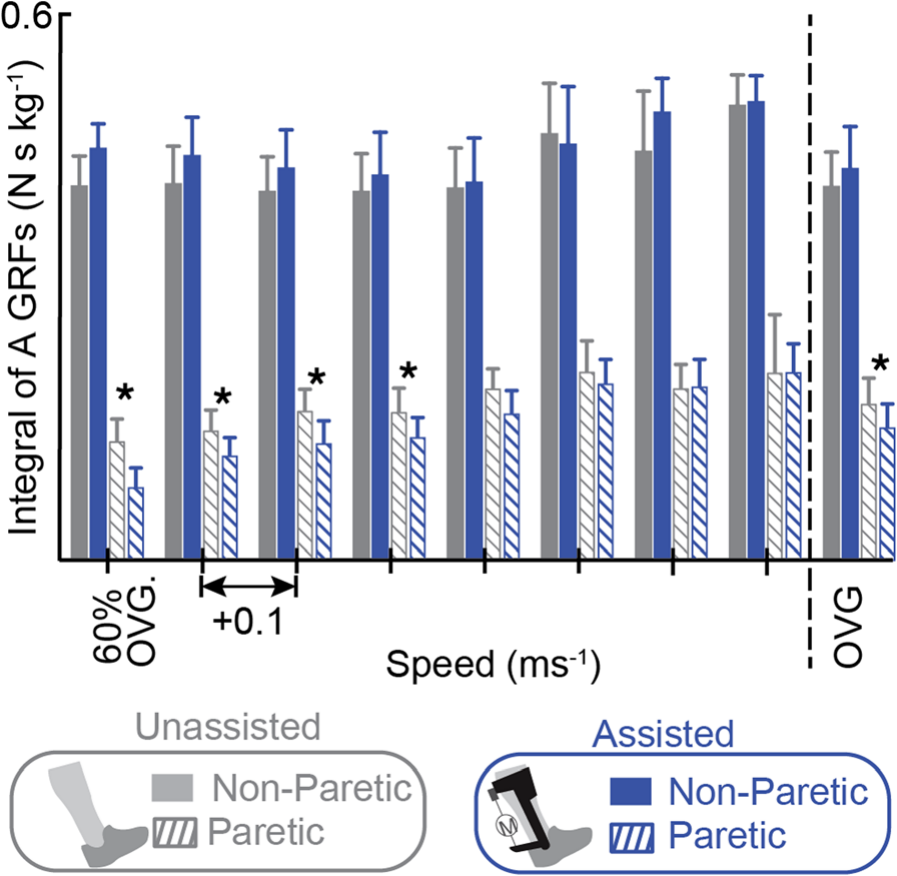
Integrated anteriorly directed GRFs on the paretic limb decreased with exoskeleton assistance at the majority of speeds. The paretic (hatch fill) and non-paretic (solid fill) integrated anterior GRFs (± standard error) are plotted with (blue) and without (grey) exoskeleton assistance applied as walking speed increases. To the right of the dashed line integrated GRFs are averaged at users’ comfortable OVG walking speed

Integrated anteriorly directed GRFs for the paretic limb were significantly lower with exoskeleton assistance when compared to the *Unassisted* condition at five speeds (*n00: p* = 0.043, *d* = 0.87; *n01: p* = 0.033, *d* = 0.87; *n02: p* = 0.007, *d* = 0.58; *n03: p* = 0.008, *d* = 0.45; *OVG: p* = 0.025, *d* = 0.38) (Fig. 4). There were no significant changes in non-paretic propulsion (Fig. 4).

### Peak vertical GRF during propulsion

**Fig. 5:**
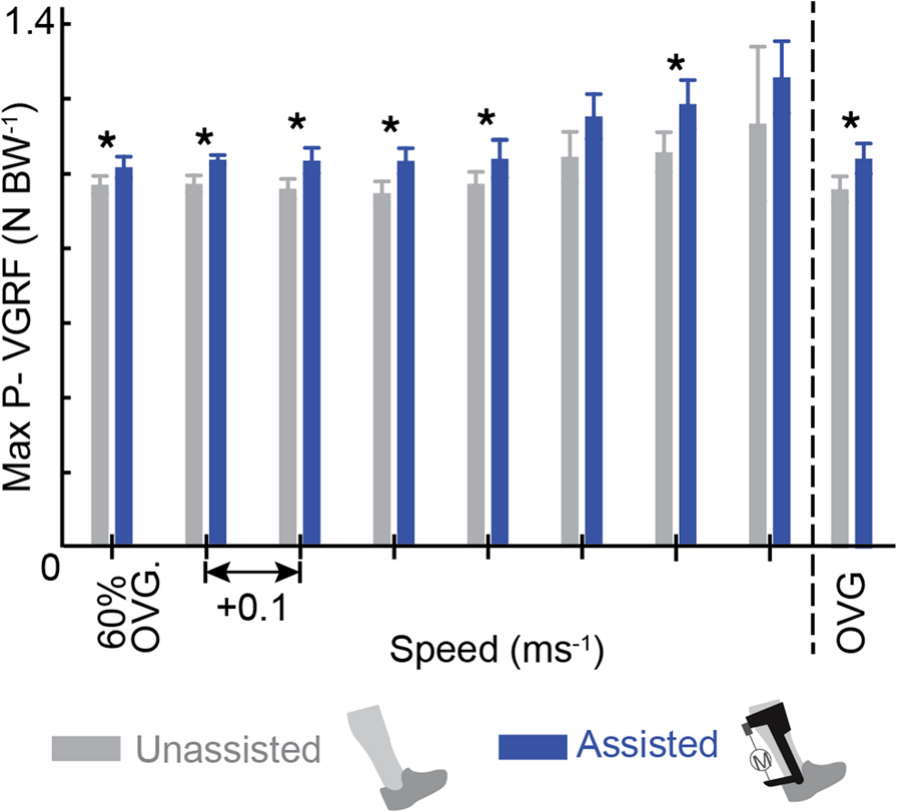
Increased paretic peak pushoff vertical GRF in the *Assisted* condition supports suggestion that reductions in TLA encourage the conversion of exoskeleton torque into vertical rather than forward propulsion. The peak paretic vertical GRF during pushoff are plotted with (blue) and without (grey) exoskeleton assistance applied as walking speed increases. To the right of the dashed line peak vertical GRF are averaged at each user’s comfortable OVG speed

During *Assisted* walking, subject averaged peak vertical GRF was increased when compared to the *Unassisted* condition at six speeds (Fig. 5), (*n00: p* = 0.026, *d* = 0.73; *n01: p* = 0.008, *d* = 1.11; *n02: p* = 0.002, *d* = 1.01; n03*: p* = 0.001, *d* = 1.075; *n04: p* = 0.001, *d* = 1.08; *n06: p* = 0.012, *d* = 0.98; *OVG: p* < 0.001, *d* = 0.89).

### TLA

**Fig. 6:**
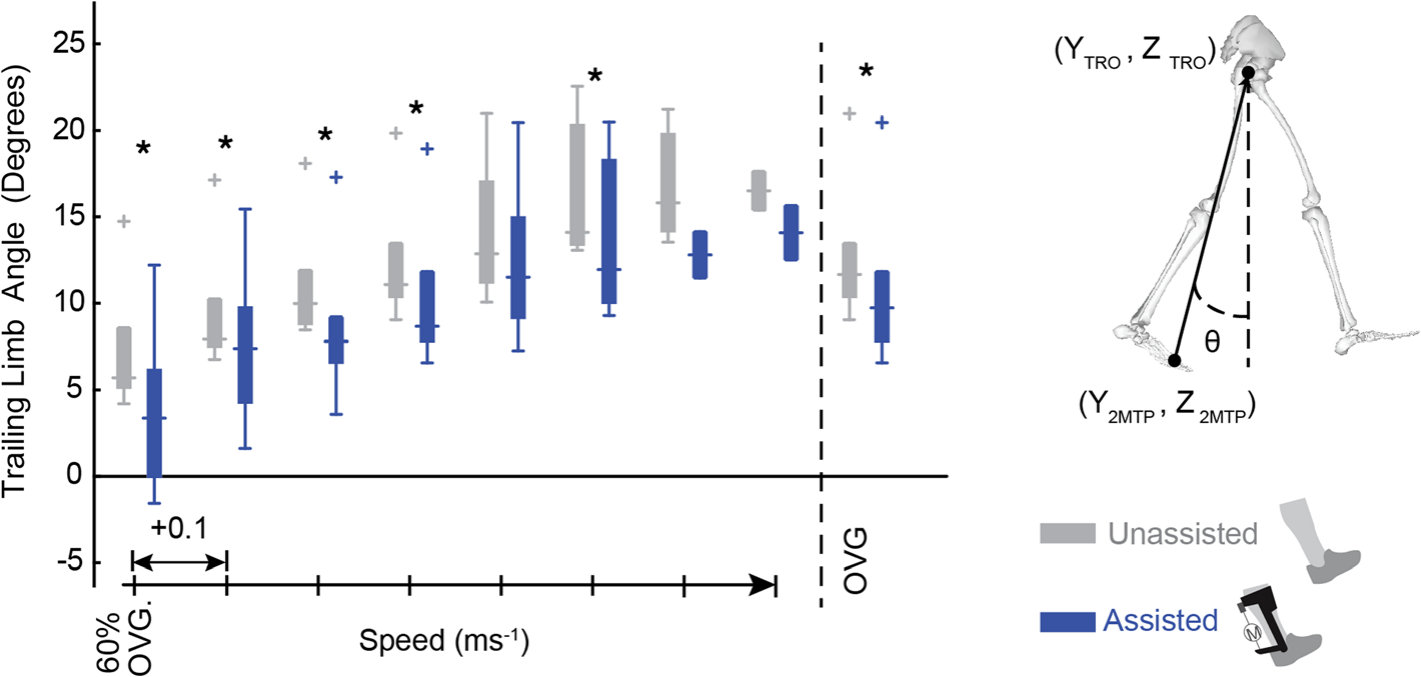
Reductions in TLA in the *Assisted* condition indicate suboptimal limb configuration during exoskeleton assistance. The paretic TLA is defined between the vertical plane and a line connecting the second Metatarsophalangeal (2MTP) joint and Greater Trochanter (TRO) during double stance. With exoskeleton assistance (blue) TLA is shown to decrease when compared to the *Unassisted* condition (grey) at all speeds. To the right of the dashed line TLA are averaged at each user’s comfortable OVG speed

During *Unassisted* walking, subject average paretic TLA increased with speed from 7.33 ° to 16.51 ° (Fig. 6). When compared to the *Unassisted* condition, the TLA was decreased with exoskeleton assistance at six speeds (*n00: p* = 0.018, *d* = 0.77; *n01: p* = 0.038, *d* = 0.58; *n02: p* = 0.006, *d* = 0.60; n03*: p* = 0.001, *d* = 0.49; *n05: p* = 0.043, *d* = 0.48; *OVG: p* = 0.002, *d* = 0.39).

## Discussion

The use of ankle-based rehabilitation strategies has increased in popularity in recent years [7–9, 11, 15, 29]. Our controller builds upon the foundation provided by Takahashi et al. through the inclusion of: (1) a speed-adaptive gain capable of scaling exoskeleton torque with walking speed and (2) an EMG adaptive gain (similar to [15]) calculated by the moving average of soleus EMG peaks over five strides to ensure the control is still saturated despite reductions in soleus EMG that can occur while using myoelectric controllers [15]. To our knowledge, this is the first study to implement a powered ankle exoskeleton that modulates plantarflexion torque magnitude with walking speed. In the current work we specifically investigated the impact of our novel controller across a range of speeds to elucidate the relationships among ankle assistance, walking speed, and walking performance for persons post-stroke. The results of this study provide a foundation for improved development of future ankle-based rehabilitation technologies capable of adapting to the user and the environment.

In support of our first hypothesis, our speed-adaptive gain performed as intended by increasing assistance with walking speed. This successful assistance modulation provides a new framework by which we can explore and interpret the impact of assistance on walking function across a range of speeds. Our second hypothesis was also supported; peak total paretic ankle power increased with exoskeleton assistance and with speed, and the exoskeleton delivered net positive energy at the paretic ankle proportional to changes in walking speed. Additionally, average net paretic limb power was increased with exoskeleton assistance, suggesting that assistance applied at the ankle transferred energy to the paretic limb as intended. Despite increases in ankle and limb power, our third hypothesis was not supported: average metabolic cost of transport showed no significant reduction with exoskeleton assistance.

Failure to convert exoskeleton assistance to forward propulsion could explain the lack of metabolic benefits seen with exoskeleton assistance in this study as previous studies have shown an inverse relationship between metabolic cost and measures of paretic propulsion [7]. Specifically, an exosuit for persons post-stroke reduced the metabolic cost of walking and was accompanied by small increases in percent paretic propulsion in addition to improved joint powers similar to the results here [7]. We expected that increased ankle power from exoskeleton assistance would yield an increase in paretic propulsion because the ankle plays a key role in forward propulsion during healthy walking [30] . Despite increases in ankle power, paretic propulsion was reduced for the *Assisted* condition compared to the *Unassisted* condition, suggesting that exoskeleton assistance at the ankle was not converted to forward propulsion. Since exoskeleton benefits were apparent in both joint and limb powers but did not translate to forward propulsion, we explored whether overall limb configuration limited the transfer of mechanical energy at the ankle into center of mass propulsion. Reductions in TLA, a commonly used measure of limb configuration, is characteristic of hemiparetic gait. Reduced TLA can further impede the transfer of power from the ankle to propulsion of the COM and reduce long-term walking function [18]. TLA values for the *Unassisted* condition reported here are within the range of TLA reported for persons post-stroke in literature [31]. In the *Assisted* condition, TLA was further reduced, bringing the trailing limb closer to vertical and apparently accelerating the COM vertically rather than anteriorly during exoskeleton assistance. Thus, while joint and limb powers were increased, conversion of ankle torque into forward propulsion was limited by suboptimal limb kinematics. The increase in peak vertical GRF during propulsion seen in the *Assisted* condition when compared to the *Unassisted* condition provides further support for the suggestion that decreased TLA encouraged conversion of exoskeleton assistance to vertical rather than forward propulsion. TLA is determined by the interactions of lower limb kinematic properties (Additional file 1: Figure S1, Additional file 2: Figure S2, Additional file 3: Figure S3, and Additional file 4: Figure S4), but it is not immediately apparent what caused the decrease in TLA for the *Assisted* condition. It is possible that the increase in ankle torque may induce limb instability, such that subjects decrease TLA during assistance as a protective mechanism to maintain stability. Future analyses could more directly examine the interaction between exoskeleton assistance and TLA. Nevertheless, the current study highlights the importance of limb configuration during exoskeleton assistance. Previous examples of biofeedback and verbal cues demonstrate their potential for improving hemiparetic gait; therefore, future research could address this concern using biofeedback or verbal cues that guide users into optimal limb configurations. Specifically, visual feedback of plantarflexor and dorsiflexor EMG signals during post-stroke walking allowed users to increase their walking speed as well as ankle power generation during the pushoff phase of gait [32]. Verbal qualitative feedback about walking performance has been shown to improve OVG walking speed [32, 33] and could be leveraged to increase TLA during exoskeleton assistance to increase propulsion. Alternative solutions to suboptimal limb configurations include investigating the timing of exoskeleton assistance or using a multi-joint exoskeleton capable of accounting for TLA during propulsion. Specifically, an exoskeleton providing assistance during the eccentric phase of soleus activity could allow greater tibial progression during stance, increasing the TLA in preparation for the assistance applied during propulsion. Alternatively, exoskeletons or robotic training aids acting across multiple joints [34–36] offer a promising tool for applying assistance and could encourage users into optimal limb configurations during pushoff.

Although altered TLA is most likely responsible for the lack of metabolic changes with exoskeleton assistance, other factors- including acclimation time and assistance timing - are known to impact energy consumption during walking [7, 13, 37]. Participants had limited acclimation to exoskeleton assistance in this study. However, previous studies of walking with powered ankle assistance indicate that in healthy subjects, gait adapts to reach steady state neuromotor and metabolic performance after ~ 30–40 min of walking practice [13]. Therefore, it is possible that increased acclimation time could improve metabolic performance. This is a challenge inherent to evaluating gait performance with exoskeleton assistance in clinical populations, for whom lengthy acclimation periods are more physically demanding and could induce fatigue. The timing and magnitude of exoskeleton assistance is known to impact metabolic costs in healthy controls [29]. Post-stroke walking performance varies markedly across individuals, thus personalized parameter settings for exoskeleton assistance may be warranted. Recent research supports this consideration, reporting for a group of persons post-stroke that personalized engagement timing when walking with an exosuit improved propulsion and reduced metabolic costs while using the device [7]. The timing of exoskeleton assistance in the current work considered the individual participant’s timing for both GRFs as well as soleus EMG signal, but it is possible that other assistance timings exist that improve torque delivery.

There are some additional limitations that should be considered. Due to the participant burden and inclusion criteria, we consider a small sample size. We did randomize the order of *Assisted* and *Unassisted* sessions for subjects; however, there was no randomization of walking speed, and therefore it is possible that at higher speeds subjects were better acclimated to exoskeleton assistance. However, as one of our goals was to see if participants could walk at faster speeds with the exoskeleton assistance, randomization of speeds was not possible. Furthermore, because each individual had a different comfortable OVG speed, evaluation speeds at each increment (n01 n02, etc.) differed in magnitude between individuals. It is also possible that 1 min was not sufficient for participants to adapt to each speed. Metabolic cost of transport was calculated across the entire ‘speed ramp’; however, this approach is subject to end effect errors because metabolic energy requirements at the end of the speed ramp may not affect measurements until sometime later. There was limited acclimation time for familiarizing the participants with exoskeleton assistance, and the exoskeleton limited the degree of freedom of the ankle to flexion. Any degree of freedom restriction caused by the physical device in other planes (e.g., inversion/eversion) is unlikely to account for differences in metabolic expenditure between the *Assisted* and *Unassisted* conditions because the exoskeleton was worn (unpowered) in the *Unassisted* condition. However, it is possible that wearing the exoskeleton limited rotations in other directions (e.g.: frontal plane) and that this limitation could affect metabolic expenditure when compared to walking without an exoskeleton. Despite this, previous exoskeletons using similar hardware have shown benefits in healthy controls, [11] and therefore, we do not believe range of motion limitations had significant negative impacts. Finally, no instructions were given to participants regarding how to optimize delivery of exoskeleton assistance.

## Conclusions

Our novel speed-adaptive proportional myoelectric controller demonstrates the potential for ankle exoskeletons to be used in rehabilitation interventions for persons post-stroke. Myoelectric controllers offer a user-controlled option for stroke rehabilitation; however, EMG data following a stroke is more variable, especially on the paretic limb where weak signals and abnormal muscle control add complications to typical processing methodology. Alternative exoskeleton controllers may mitigate some of the challenges of implementing proportional myoelectric exoskeletons outside the lab. For example, an impedance-based controller capable of reducing metabolic cost of intact human walking [11] in healthy controls offers an exciting research area for stroke rehabilitation where human-robot interaction dynamics may be tailored to the individual’s physiology. Future studies implementing impedance-based controllers and incorporating verbal cues that guide users into optimal limb configurations could exceed the capabilities of the current work and contribute to reduced metabolic cost of transport for persons post-stroke walking with an ankle exoskeleton.

## Additional file

Additional file 1: **Figure S1.** Ankle, knee and hip joint angles are shown for the non-paretic and paretic limbs with and without exoskeleton assistance. Joint angles are calculated from subject averages and are plotted with percent stride for all walking speeds. (PNG 525 kb)

Additional file 2: **Figure S2.** Ankle, knee and hip joint velocities are shown for the non-paretic and paretic limbs with and without exoskeleton assistance. Joint velocities are calculated from subject averages and are plotted with percent stride for all walking speeds. (PNG 546 kb)

Additional file 3: **Figure S3.** Ankle, knee and hip joint moments are shown for the non-paretic and paretic limbs with and without exoskeleton assistance. Joint Moments are calculated from subject averages and are plotted with percent stride for all walking speeds. Exoskeleton torque (dashed) is plotted with total paretic ankle moment (solid). (PNG 470 kb)

Additional file 4: **Figure S4.** Ankle, knee and hip joint powers are shown for the non-paretic and paretic limbs with and without exoskeleton assistance. Joint powers are calculated from subject averages and are plotted with percent stride for all walking speeds. (PNG 439 kb)

Additional file 5: **Figure S5.** Average positive joint powers presented as percentages of total joint contributions demonstrate the largest impact of exoskeleton assistance is increases in total (biological + exoskeleton) ankle power at five speeds when compared to the *Unassisted* condition. Rows of pie charts represent walking speed starting at n00 and increasing until the horizontally dashed line; the top row of pie charts represents positive average joint contributions at comfortable OVG speed. Pie charts represent the ankle (blue), knee (green), and hip (orange) contributions and are organized in the following columns (from left to right): 1) non-paretic joints *Unassisted* (light), 2) non-paretic joints *Assisted* (dark), 3) paretic joints *Unassisted* (light), and 4) paretic joints *Assisted* (dark). The diameter of each pie is scaled by the maximum sum of average positive joint powers (n07, non-paretic, *Unassisted*). Paired t-tests were calculated according to values of average positive joint powers rather than the contribution of a joint to the summed joint powers. Paretic: Total (biological + exoskeleton) positive paretic ankle power was significantly higher at four speeds (*n00: p* = 0.038, *d* = 1.78; *n02: p* = 0.015, *d* = 1.97; *n03: p* = 0.018, *d* = 1.73; *n04: p* = 0.009, *d* = 2.27) as well as at comfortable OVG speed (*p* = 0.007, *d* = 1.46) with exoskeleton assistance. Paretic average biological ankle power was increased significantly at one speed (*n02*: *p* = 0.047, *d* = 1.28) with exoskeleton assistance. Lastly, average positive hip power was increased significantly at one speed (*n00*: *p* = 0.034, *d* = 1.18). No significant change was found in paretic average positive knee power at any speed. Non-Paretic: Average positive non-paretic ankle power increased with exoskeleton assistance at two speeds (*n02*: *p* = 0.023, *d* = 0.42; *n03*: *p* = 0.012, *d* = 0.47), and average positive non-paretic knee power decreased at one speed (*n05: p* = 0.044, *d* = 0.426). (PNG 120 kb)

Additional file 6: **Figure S6.** Average negative paretic joint powers showed limited changes with exoskeleton assistance across walking speeds. Pie charts are organized by speed; the first row of pie charts is calculated at 60% of each users comfortable OVG speed (n00) and speed increases each row until the dashed line. After the dashed line average negative joint powers are calculated at each user’s comfortable OVG speed. Pie charts represent the ankle (blue), knee (green), and hip (orange) contributions and are organized by the following columns (from left to right): 1) non-paretic joints *Unassisted* (light), 2) non-paretic joints *Assisted* (dark), 3) paretic joints *Unassisted* (light), and 4) paretic joints *Assisted* (dark). Note that the diameters are scaled by dividing the sum of joint contributions for each pie by the maximum sum of average positive joint powers (n07, non-paretic, *Unassisted*). Although the pie charts illustrate percentage contributions from each joint t-tests were performed by comparing values for average negative joint power for the *Unassisted* and *Assisted* conditions. Paretic: The magnitude of average negative knee powers were increased at two speeds for the *Assisted* when compared to the *Unassisted* condition (n05: *p* = 0.044, *d* = 0.76; *OVG*: *p* = 0.031, *d* = 0.47). Non-paretic: The magnitude of average negative ankle power increased at one speed for the *Assisted* when compared to the *Unassisted* condition (*n03*: *p* = 0.026, *d* = 0.74). At a different speed, the magnitude of average negative knee powers increased for the *Assisted* when compared to the *Unassisted* condition (*OVG: p* = 0.040, *d* = 0.68). (PNG 86 kb)

Additional file 7: **Figure S7.** Average net non-paretic knee power was significantly reduced at three walking speeds (*n00*: *p* = 0.045, *d* = 0.50; *n04*: *p* = 0.030, *d* = 0.60; *OVG*: *p* = 0.014, *d* = 0.60). Average net non-paretic ankle (A), knee (B), and hip (C) power (± standard error) for the *Unassisted* (light colors) and *Assisted* (dark colors) conditions. All values are calculated from subject averages over five gait cycles. To the right of the dashed line average net powers averaged at each user’s comfortable OVG speed are shown. (PNG 74 kb)

Additional file 8: **Figure S8.** Mass normalized metabolic power during each participant’s *Assisted* and *Unassisted* data collection sessions. The normalized metabolic power is plotted for participants one (A), two (B), three (C), four (D), five (E), and six (F). Fit lines were generated based on a second order polynomial. (PNG 654 kb)

## Abbreviations

EMG: Electromyography
GRF: Ground reaction force
IRB: Institutional review board
OVG: Overground
TLA: Trailing limb angle
