# Supplementary figures and images for: Mechanics and energetics of post-stroke walking aided by a powered ankle exoskeleton with speed-adaptive myoelectric control
Source: J Neuroeng Rehabil. 2019 May 15;16:57. doi: 10.1186/s12984-019-0523-y (PMC6521500; doi:10.1186/s12984-019-0523-y)

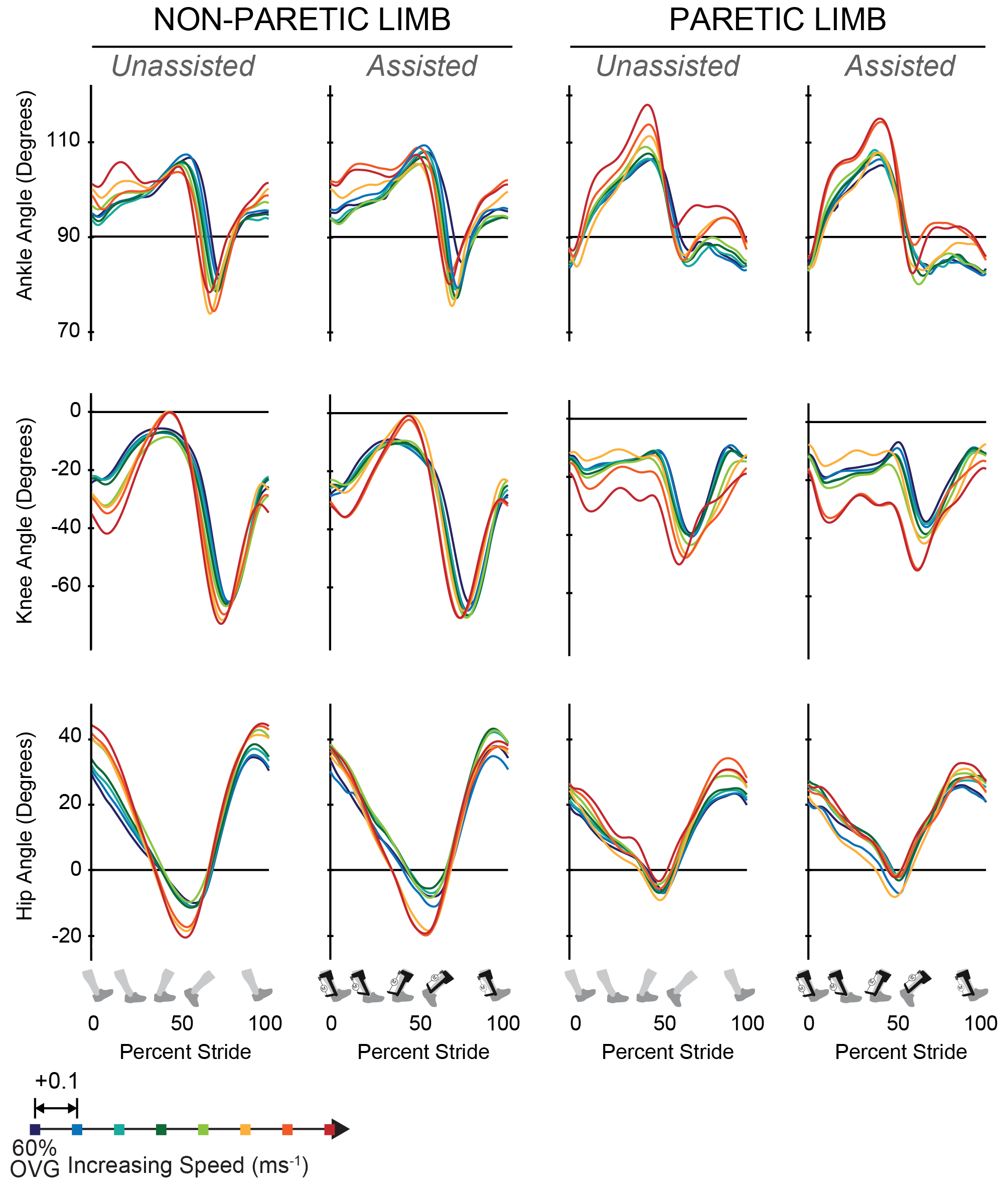

Supplement: Supplementary file 1 — Figure S1. Ankle, knee and hip joint angles are shown for the non-paretic and paretic limbs with and without exoskeleton assistance. Joint angles are calculated from subject averages and are plotted with percent stride for all walking speeds. (PNG 525 kb) [file 12984_2019_523_MOESM1_ESM.png]

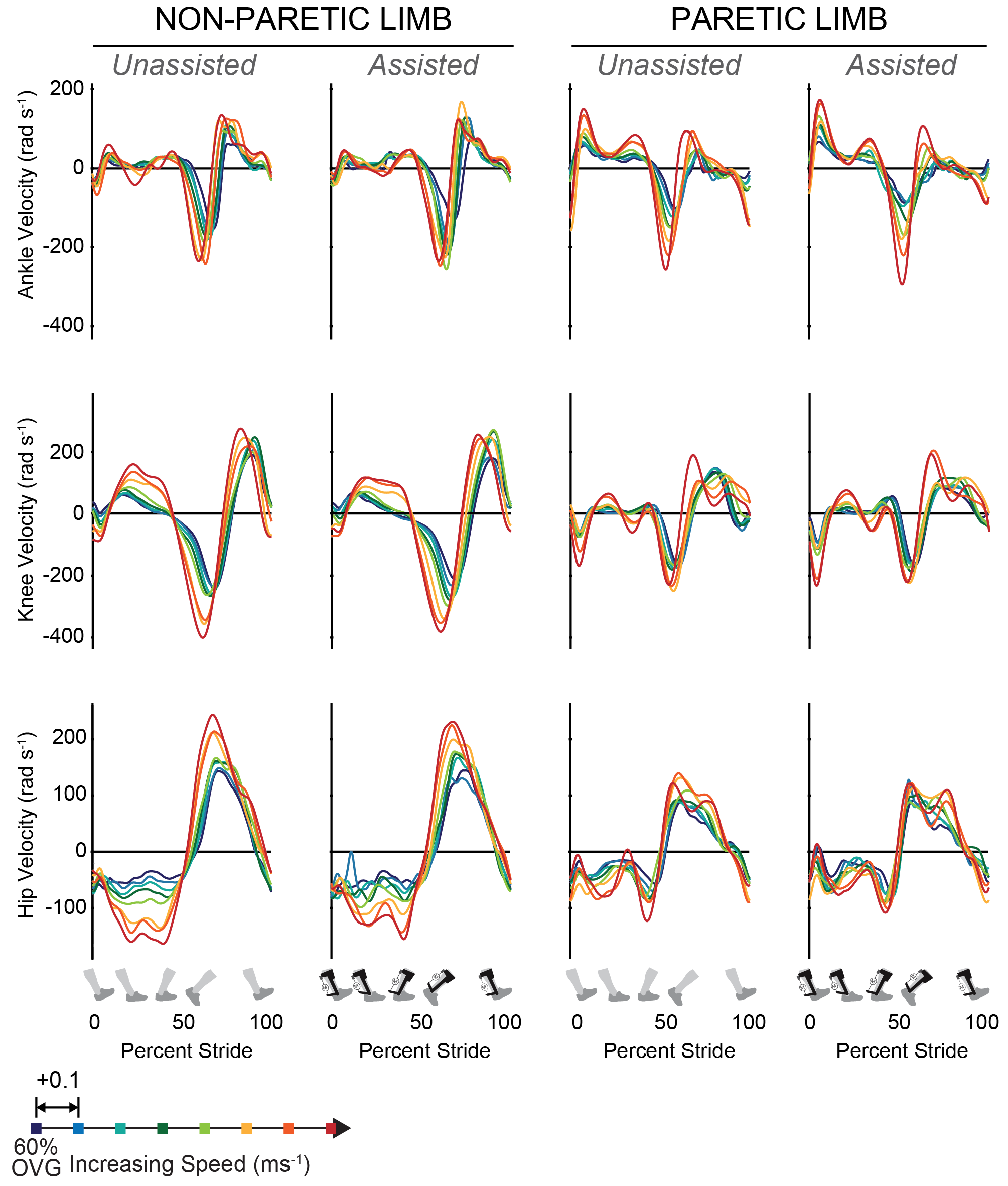

Supplement: Supplementary file 2 — Figure S2. Ankle, knee and hip joint velocities are shown for the non-paretic and paretic limbs with and without exoskeleton assistance. Joint velocities are calculated from subject averages and are plotted with percent stride for all walking speeds. (PNG 546 kb) [file 12984_2019_523_MOESM2_ESM.png]

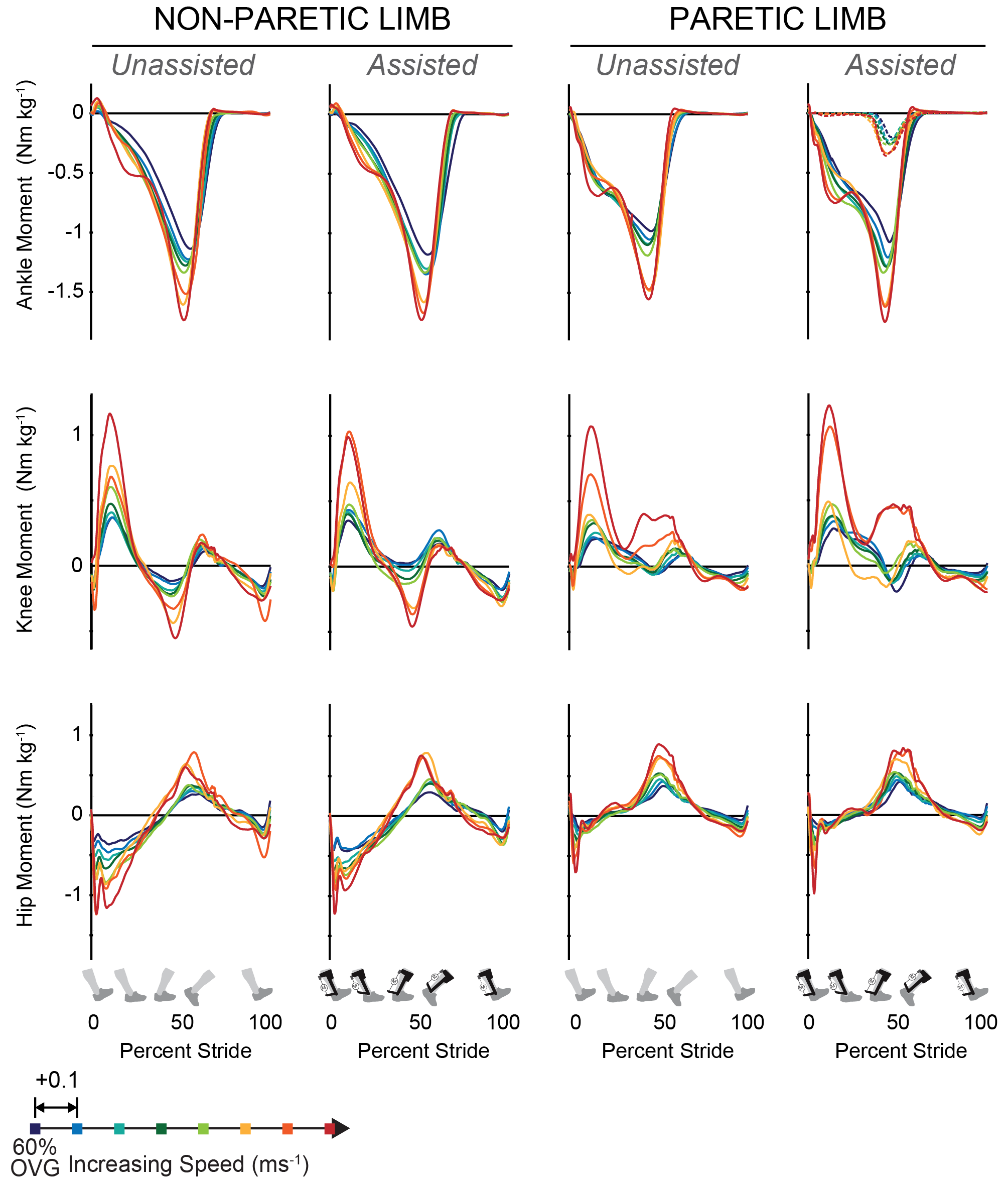

Supplement: Supplementary file 3 — Figure S3. Ankle, knee and hip joint moments are shown for the non-paretic and paretic limbs with and without exoskeleton assistance. Joint Moments are calculated from subject averages and are plotted with percent stride for all walking speeds. Exoskeleton torque (dashed) is plotted with total paretic ankle moment (solid). (PNG 470 kb) [file 12984_2019_523_MOESM3_ESM.png]

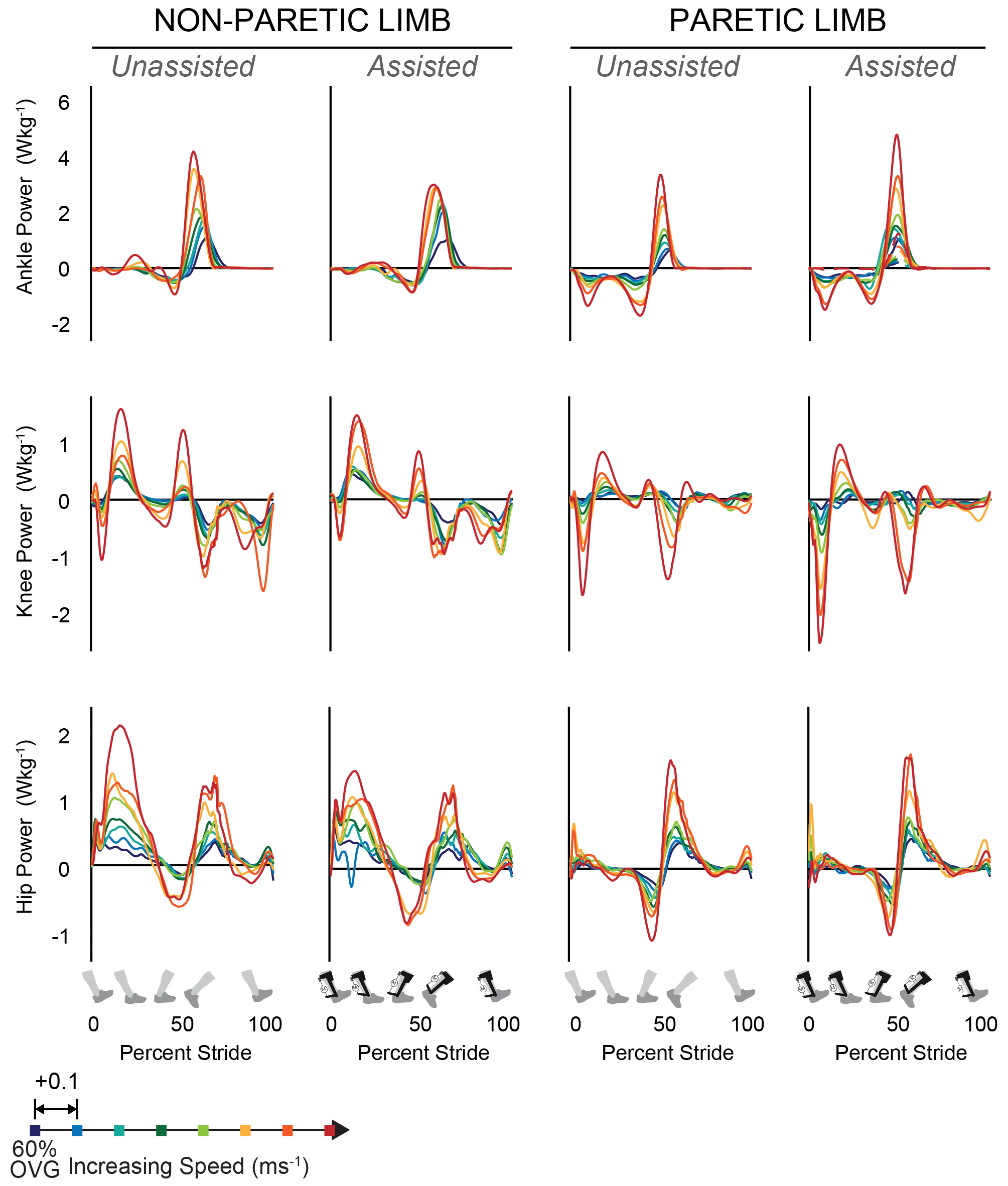

Supplement: Supplementary file 4 — Figure S4. Ankle, knee and hip joint powers are shown for the non-paretic and paretic limbs with and without exoskeleton assistance. Joint powers are calculated from subject averages and are plotted with percent stride for all walking speeds. (PNG 439 kb) [file 12984_2019_523_MOESM4_ESM.png]

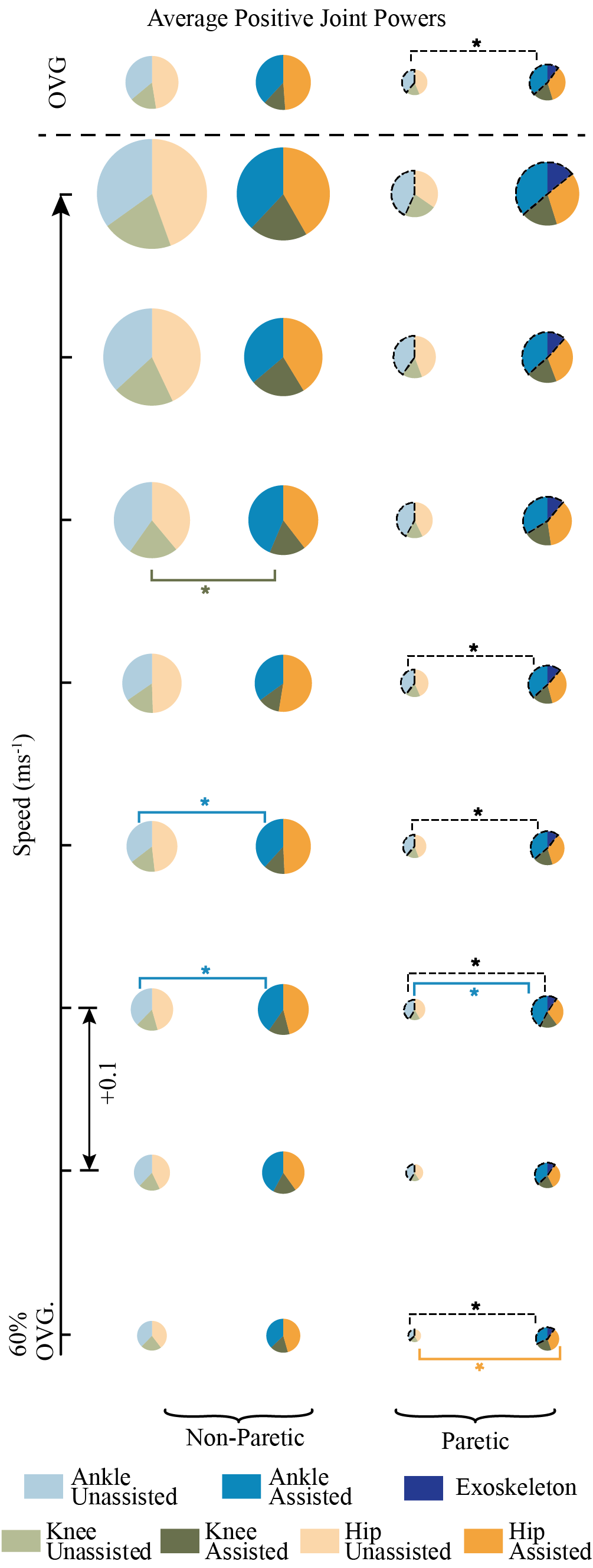

Supplement: Supplementary file 5 — Figure S5. Average positive joint powers presented as percentages of total joint contributions demonstrate the largest impact of exoskeleton assistance is increases in total (biological + exoskeleton) ankle power at five speeds when compared to the Unassisted condition. Rows of pie charts represent walking speed starting at n00 and increasing until the horizontally dashed line; the top row of pie charts represents positive average joint contributions at comfortable OVG speed. Pie charts represent the ankle (blue), knee (green), and hip (orange) contributions and are organized in the following columns (from left to right): 1) non-paretic joints Unassisted (light), 2) non-paretic joints Assisted (dark), 3) paretic joints Unassisted (light), and 4) paretic joints Assisted (dark). The diameter of each pie is scaled by the maximum sum of average positive joint powers (n07, non-paretic, Unassisted). Paired t-tests were calculated according to values of average positive joint powers rather than the contribution of a joint to the summed joint powers. Paretic: Total (biological + exoskeleton) positive paretic ankle power was significantly higher at four speeds (n00: p = 0.038, d = 1.78; n02: p = 0.015, d = 1.97; n03: p = 0.018, d = 1.73; n04: p = 0.009, d = 2.27) as well as at comfortable OVG speed (p = 0.007, d = 1.46) with exoskeleton assistance. Paretic average biological ankle power was increased significantly at one speed (n02: p = 0.047, d = 1.28) with exoskeleton assistance. Lastly, average positive hip power was increased significantly at one speed (n00: p = 0.034, d = 1.18). No significant change was found in paretic average positive knee power at any speed. Non-Paretic: Average positive non-paretic ankle power increased with exoskeleton assistance at two speeds (n02: p = 0.023, d = 0.42; n03: p = 0.012, d = 0.47), and average positive non-paretic knee power decreased at one speed (n05: p = 0.044, d = 0.426). (PNG 120 kb) [file 12984_2019_523_MOESM5_ESM.png]

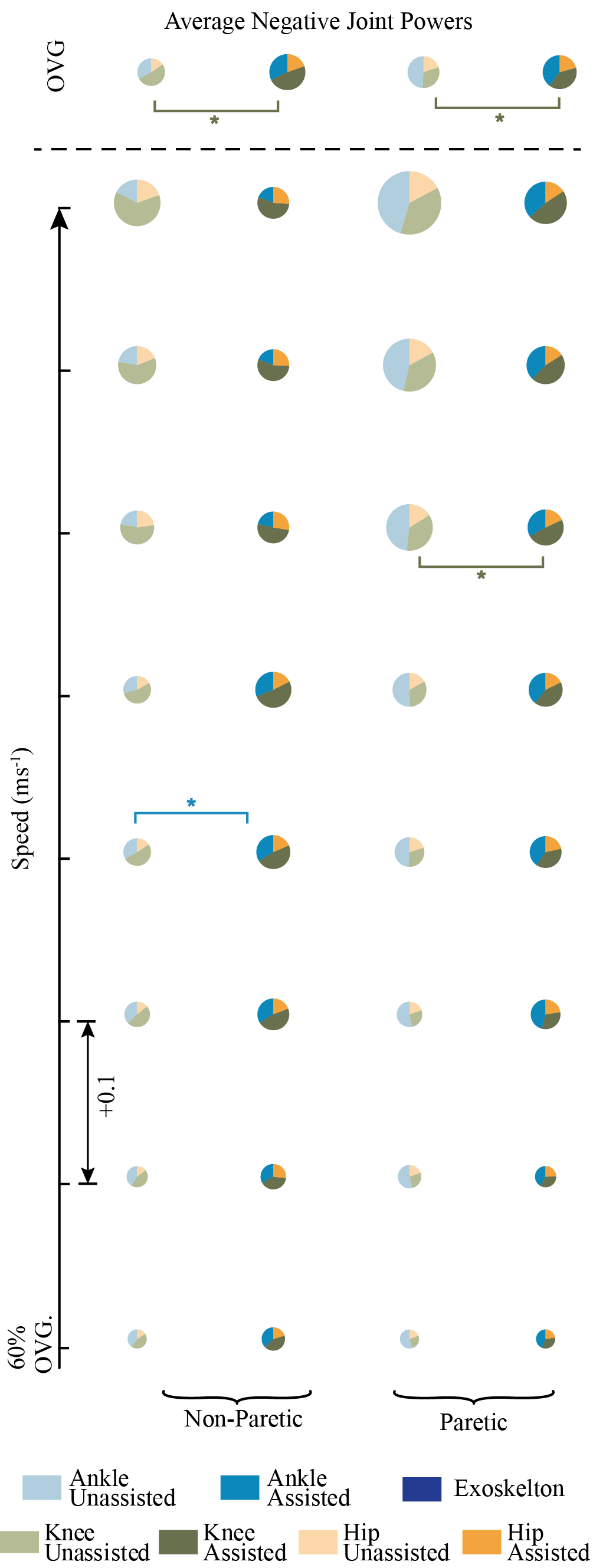

Supplement: Supplementary file 6 — Figure S6. Average negative paretic joint powers showed limited changes with exoskeleton assistance across walking speeds. Pie charts are organized by speed; the first row of pie charts is calculated at 60% of each users comfortable OVG speed (n00) and speed increases each row until the dashed line. After the dashed line average negative joint powers are calculated at each user’s comfortable OVG speed. Pie charts represent the ankle (blue), knee (green), and hip (orange) contributions and are organized by the following columns (from left to right): 1) non-paretic joints Unassisted (light), 2) non-paretic joints Assisted (dark), 3) paretic joints Unassisted (light), and 4) paretic joints Assisted (dark). Note that the diameters are scaled by dividing the sum of joint contributions for each pie by the maximum sum of average positive joint powers (n07, non-paretic, Unassisted). Although the pie charts illustrate percentage contributions from each joint t-tests were performed by comparing values for average negative joint power for the Unassisted and Assisted conditions. Paretic: The magnitude of average negative knee powers were increased at two speeds for the Assisted when compared to the Unassisted condition (n05: p = 0.044, d = 0.76; OVG: p = 0.031, d = 0.47). Non-paretic: The magnitude of average negative ankle power increased at one speed for the Assisted when compared to the Unassisted condition (n03: p = 0.026, d = 0.74). At a different speed, the magnitude of average negative knee powers increased for the Assisted when compared to the Unassisted condition (OVG: p = 0.040, d = 0.68). (PNG 86 kb) [file 12984_2019_523_MOESM6_ESM.png]

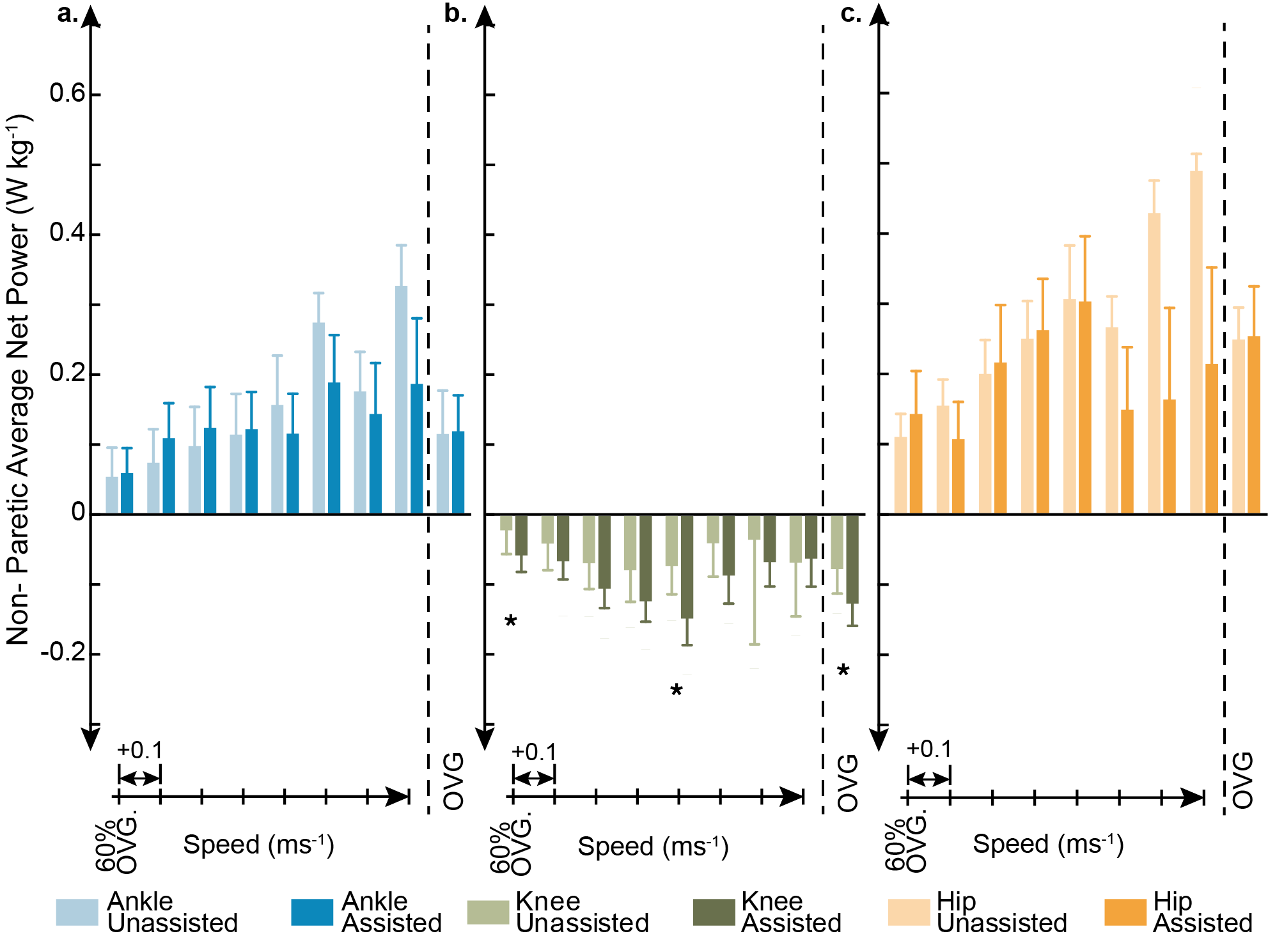

Supplement: Supplementary file 7 — Figure S7. Average net non-paretic knee power was significantly reduced at three walking speeds (n00: p = 0.045, d = 0.50; n04: p = 0.030, d = 0.60; OVG: p = 0.014, d = 0.60). Average net non-paretic ankle (A), knee (B), and hip (C) power (± standard error) for the Unassisted (light colors) and Assisted (dark colors) conditions. All values are calculated from subject averages over five gait cycles. To the right of the dashed line average net powers averaged at each user’s comfortable OVG speed are shown. (PNG 74 kb) [file 12984_2019_523_MOESM7_ESM.png]

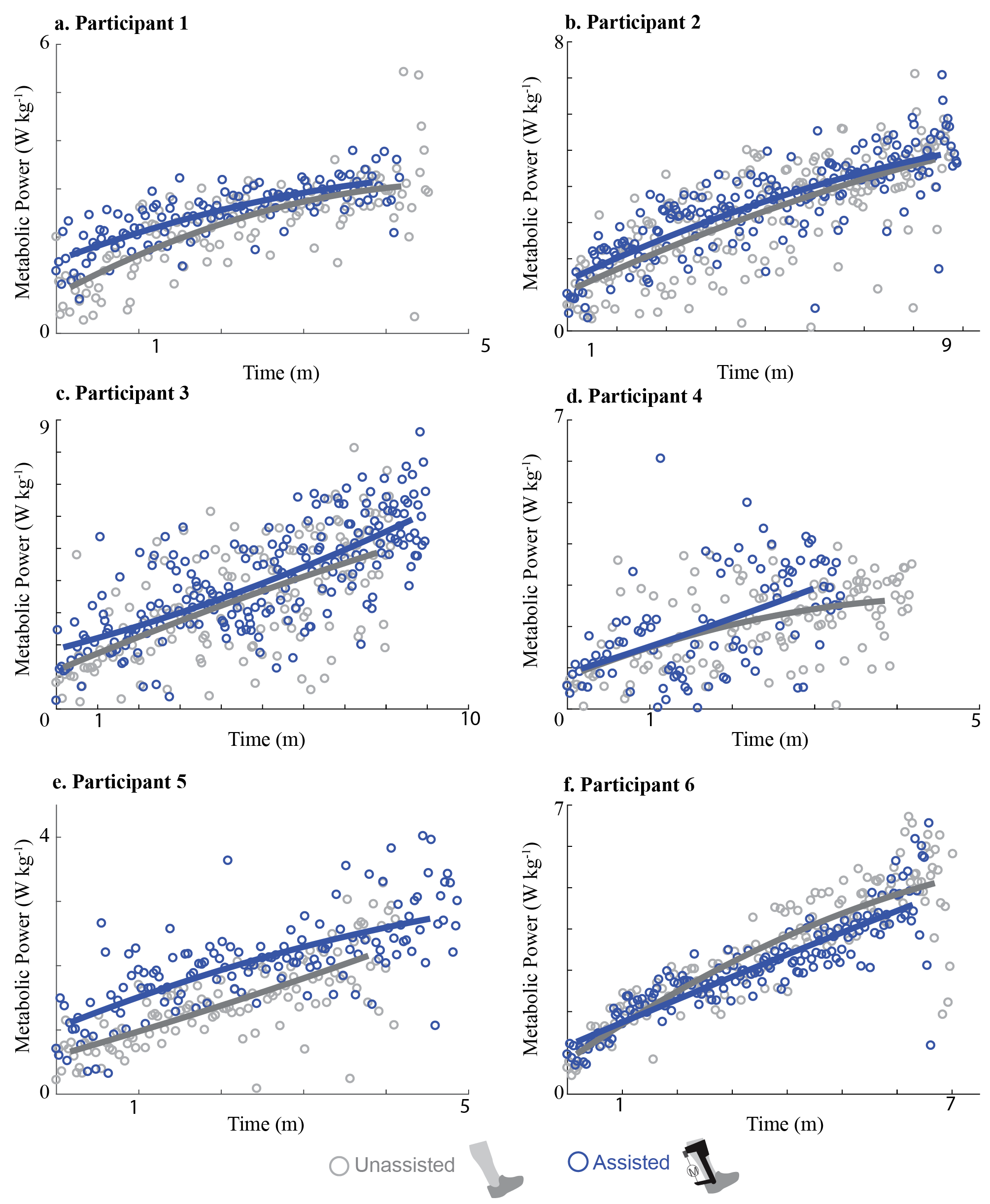

Supplement: Supplementary file 8 — Figure S8. Mass normalized metabolic power during each participant’s Assisted and Unassisted data collection sessions. The normalized metabolic power is plotted for participants one (A), two (B), three (C), four (D), five (E), and six (F). Fit lines were generated based on a second order polynomial. (PNG 654 kb) [file 12984_2019_523_MOESM8_ESM.png]
